# The Impact of Sleeve Gastrectomy on Females With Polycystic Ovarian Syndrome From 2018 to 2020 in Riyadh, Saudi Arabia: A Prospective Study

**DOI:** 10.7759/cureus.10284

**Published:** 2020-09-06

**Authors:** Alhanouf F Altamimi, Zuhour A Alqahtani, Kholoud A Alshiha, Fay Almughaiseeb, Norah F Alfayez, Alexandra A Alkhatir

**Affiliations:** 1 Medicine, Imam Mohammad Ibn Saud Islamic University, Riyadh, SAU; 2 Obstetrics and Gynaecology, Imam Mohammad Ibn Saud Islamic University, Riyadh, SAU

**Keywords:** polycystic ovarian syndrome, sleeve gastrectomy, bariatric surgery, infertility

## Abstract

Background: Obesity is one of the most common risk factors for polycystic ovarian syndrome (PCOS). Reducing body weight has shown to improve the symptoms of PCOS; however, it is still unclear if the surgical treatment of obesity can have better outcomes to control obesity and improve PCOS.

Objective: This study aims to identify the impact of sleeve gastrectomy on PCOS symptoms in Riyadh, Saudi Arabia with a comparison of other weight reduction surgeries.

Design and Setting: This is a prospective study that included patients with PCOS who had weight reduction surgeries between 2018 and 2020, in Riyadh, Saudi Arabia. Using an online questionnaire, demographic data will be collected and information about menstruation, abortion, polycystic ovarian syndrome, and bariatric surgeries (if any). Data analysis was done through Statistical Package for the Social Sciences (SPSS) v26 (IBM Corp., Armonk, NY, USA).

Results: Ninety-nine female patients responded to this survey, all of whom had PCOS; 57.6% of these patients had a positive family history of PCOS. The most-reported PCOS symptoms were irregular menses (63.6%) and weight gain (53.5%), while 9.1% were asymptomatic. 52.5% were obese, and 91.1% underwent gastric sleeve surgery to treat their obesity. 41.4% of the females had a regular cycle before the operation, which increased to 60.6% after the operation; 48.5% had a normal flow before the operation, which increased to 61.6% after the operation. 15.2% conceived once before the operation, which increased to 16.2% after the operation. 11.1% had one abortion before surgery, which decreased to 7.1% after surgery. 55.6% observed an improvement in their PCOS symptoms after surgery, and 47.5% found that their fertility improved according to their own perception. Females with a positive family history of PCOS had a higher prevalence of PCOS (p-value <0.001). Also, females 30-39 years old are significantly more likely to have PCOS (p-value <0.001). The incidence of abnormal menstrual flow and irregular menstrual cycle was seen significantly more in PCOS patients than non-PCOS (p-value=0.019 and 0.004, respectively). PCOS symptoms and fertility significantly improved after surgery (p-value=0.031 and 0.043, respectively).

Conclusion: Sleeve gastrectomy can lead to significant improvement in fertility and symptoms of PCOS. It can also reduce the incidence of abortion. Other surgical techniques should be investigated.

## Introduction

Polycystic ovarian syndrome (PCOS) is a common condition globally [1]. It is more prevalent in young adult and middle-aged females. PCOS can significantly affect a female’s fertility and can increase the incidence of abortion [2]. Additionally, the most common risk factors for PCOS are obesity and insulin resistance [3].

PCOS is associated with hyperandrogenism, ovulatory disorder, polycystic ovaries, and significant thyroid dysfunction. PCOS can increase the risk of endometrial hyperplasia or cancer, diabetes, and cardiovascular disease [4]. The etiology of PCOS is still unknown; however, it is frequently correlated to metabolic syndromes [5].

Treatment of PCOS is primarily dependent on lifestyle modification and medications in some patients [6]. The lifestyle modifications aim to reduce body weight, which can lead to significant improvement in insulin sensitivity, and consequently, the symptoms of PCOS [7]. Metformin is the most commonly used medication and it also targets improving insulin sensitivity, in addition to managing obesity [8].

Obesity is defined as an increased body weight which results in a body mass index (BMI) of ≥30 kg/m2 [5]. In Saudi Arabia, the prevalence of obesity is increasing among both genders and in all age groups [9]. Consequently, obesity increases the risk of Saudi females getting PCOS and its complications. It can increase the risk of menstrual disorders and fertility [5]. Obesity is also more common in patients who have other endocrine disorders. Obesity can be treated through lifestyle modifications, medications, and surgical treatment [10].

Although controlling obesity can improve PCOS symptoms, it is still unclear which treatment strategy for obesity can have the best effect on PCOS [11]. Until present, there is no data about the influence of sleeve gastrectomy operations on the symptoms of PCOS [12]. Therefore, the goal of this investigation is to explore the effect of a gastric sleeve on the control of obesity and thus improving PCOS symptoms in Riyadh, Saudi Arabia.

## Materials and methods

Study design

This is a prospective cohort study in Riyadh, Saudi Arabia. Done through an online questionnaire developed especially for this study to assess the result of weight reduction surgeries on PCOS patients, the questionnaire was given to females of Saudi nationality who underwent weight reduction surgeries (mainly sleeve gastrectomy) and have PCOS. Their ages were from 15 years old (onset of puberty) to 51 years old (menopause). Any participant who did not match these inclusion criteria was excluded.

Data collection

An online self-developed questionnaire was sent electronically to female patients. The survey included demographic data, questions regarding PCOS and its effect on the patient, questions regarding the surgery and the post-surgical aspect of PCOS, in addition to menstrual disorders and pregnancy complications. All the information and medical history were collected from the questionnaire, not from any medical record.

Statistical analyses

A convenient sample of Saudi post-sleeve gastrectomy females was included. Data was represented in terms of frequencies and valid percentages for categorical variables, and compared using the Fisher's exact test. For continuous variables, it was described as mean (standard deviation). All p-values <0.05 were considered statistically significant. IBM Statistical Package for the Social Science (SPSS; IBM Corp., Armonk, NY, USA) was used to perform all statistical calculations with version 26 for Microsoft Windows.

Ethical considerations

Institutional research ethics board approval was acquired before conducting any study procedure. Participants were informed that participation is voluntary and that they have the right to withdraw from the study at any time and were assured that all their information will be kept anonymous and confidential.

## Results

Ninety-nine female patients responded to this online questionnaire in this study. Only participants who fit the inclusion criteria were included. Socio-demographics of participants and analysis of the questionnaire are shown below.

General characters of responders

Out of 99 female participants, age was sub-categorized into five age groups, starting from 20 years to 51. The most prevalent age group was between 20 to 29 years old (42.4%). As for marital status, 53.5% of the females were married. The area of residence was classified into middle, western, eastern, southern, and northern. 71.7% of the females were from the middle area. All socio-demographic data is shown in detail in Table 1.

**Table 1:** Socio-demographic data of responders to the questionnaire.

		Count	Percent
Age group	20-29	42	42.4
	30-39	38	38.4
	40-49	14	14.1
	50-51	5	5.1
Marital Status	Single	35	35.4
	Married	53	53.5
	Did not mention	11	11.1
Region	Eastern	3	3
	Middle	71	71.7
	Northern	2	2
	Southern	12	12.1
	Western	11	11.1

Diagnosis and history of PCOS

Participants were asked about their PCOS diagnosis and their family history for PCOS. The majority of menarche age between participants was from 11 to 13 years old (62.6%), and the average duration for menstruation was 5.96±0.684 days. 11.1% passed through menopause at the time of the questionnaire, while only 4% of participants were at menopause (based on the subjective opinion of the respondents). All the patients included in this study had a diagnosis of PCOS, and 57.6% of them had a positive family history of PCOS.

Symptoms of PCOS were difficult pregnancy, hirsutism, irregular menses, thinning of hair or hair loss, weight gain, and more than one symptom. Nine percent of the participants were asymptomatic, as shown in Table 2.

**Table 2:** polycystic ovarian syndrome (PCOS) history and diagnosis

		Count	Percent
Age of menarche	9-10 years	10	10.1
	11-13 years	62	62.6
	14-16 years	24	24.2
	17-20 years	3	3
Have you passed through menopause	Yes	11	11.1
	No	88	88.9
Are you currently in menopause	Yes	4	4
	No	75	75.8
	Did not mention	20	20.2
Family history of PCOS	Yes	57	57.6
	No	31	31.3
	Did not mention	11	11.1
Symptoms of PCOS	Asymptomatic	9	9.1
	Difficult pregnancy	30	30.3
	Hirsutism	45	45.5
	Irregular menses	53	53.5
	Thinning of hair or hair loss	34	34.3
	Weight gain	63	63.6
	Did not mention	11	11.1

Participants were also asked if they had any other medical conditions. 29.2% of the females did not have any other medical conditions, while 52.5% were obese; the assessment of obesity was based on the patient’s perception. 14.1% of the other participants had thyroid disease.

Management of obesity

Patients responded to a question about the treatment strategy that they used to treat their obesity (pharmacological, non-pharmacological, or surgical). Only 2% of the patients did not undergo an operation, while 91.1% underwent a gastric sleeve to treat their obesity, as shown in Table 3.

**Table 3:** Methods of treating obesity.

		Count	Percent
What type of Bariatric Surgery have you performed?
	Gastric sleeve	91	91.1
	Roux-en-Y gastric bypass	3	3
	Laparoscopic adjustable gastric banding	3	3
	Gastric Balloon	0	0

Menstruation before and after surgery

Patients were asked about their menstrual cycle regularity and flow before and after surgery. It has been shown that 41.1% of the females had a regular cycle before the operation, which increased to 60.6% after the operation. As for the menstrual flow, 48.5% of the females had a normal flow before the operation, which increased to 61.1% after the operation, as shown in Table 4

**Table 4:** Menstruation before and after surgery

		Before Surgery		After surgery	
		Count	Percent	Count	Percent
Was your menstrual cycle	Irregular	47	47.5	20	20.2
	Regular	41	41.4	60	60.6
	Did not mention	11	11.1	19	19.2
Was your menstrual flow	Heavy	17	17.2	9	9.1
	Light	23	23.2	10	10.1
	Normal	48	48.5	61	61.6
	Did not mention	11	11.1	19	19.2

Pregnancy and fertility before and after surgery

Patients were asked about their pregnancy and fertility before and after surgery. As for the number of conceptions, 15.2% of the females conceived once before the operation, which slightly increased to 16% after the operation. The frequency of experiencing abortion once decreased by 4% before and after surgery. Additionally, 6.1% of the females were infertile before the operation based on their judgement, as shown in Table 5.

**Table 5:** Pregnancy and fertility before and after surgery.

		Before Surgery		After surgery	
		Count	Percent	Count	Percent
How many times have you conceived	Once	15	15.2	16	16.2
	Twice	8	8.1	6	6.1
	Third	6	6.1	2	2
	Four-time	9	9.1	0	0
	Five or more	7	7.1	0	0
	Never	43	43.4	56	56.6
	Did not mention	11	11.1	19	19.2
How many times have you aborted?	Once	11	11.1	7	7.1
	Twice	7	7.1	2	2
	Third	1	1	0	0
	Four-time	0	0	0	0
	Five or more	1	1	0	0
	Never	68	68.7	71	71.7
	Did not mention	11	11.1	19	19.2
are you infertile	Yes	42	42.4	-	-
	No	6	6.1	-	-
	May be	24	24.2	-	-
	I do not know	16	16.2	-	-
	Did not mention	11	11.1	-	-

Other postoperative medical changes

Furthermore, 55.6% of the females observed an improvement in their PCOS symptoms after surgery; 47 % of the whole cohort found that their fertility had improved. As for the postoperative complications, most of the participants (43.4%) did not have any complications. The others had bleeding, gall stones, infection, and embolism. The most common complication was gall stones, occurring in 17.1% of patients, as shown in Table 6.

**Table 6:** Other postoperative medical changes. PCOS: polycystic ovarian syndrome

		Count	Percent
Has the PCOS been treated? or have the symptoms improved	Yes	55	55.6
	No	24	24.2
	Did not mention	20	20.2
Have your fertility improved	Yes	47	47.5
	No	31	31.1
	Did not mention	21	21.2
Did you have any complications after surgery	Bleeding	2	2
	Gall stones	17	17.1
	Infection	3	3
	Embolism	1	1
	Others	6	6.1
	None	43	43.4
	Did not mention	33	33.3

## Discussion

Polycystic ovarian syndrome (PCOS) is a common medical problem among females, which can reduce their quality of life significantly [13]. PCOS can reduce female fertility in addition to increased incidence of menstrual irregularities. Females with PCOS are commonly obese; hence, controlling body weight has been proposed to improve the symptoms of PCOS [14]. However, outcomes of surgical treatment of obesity using gastric sleeves are still controversial in patients with PCOS [15].

The present investigation aimed to evaluate the impact of sleeve gastrectomy on the symptoms of PCOS in Riyadh, Saudi Arabia. The study revealed that in all of the patients who had PCOS, 57.6% of them had a positive family history of PCOS. Patients with PCOS had more than one symptom of PCOS, while 9.1% were asymptomatic. Females who have a positive family history of PCOS had a higher prevalence of PCOS (p-value <0.001). As for obesity, almost half of the participants were obese (52.5%), and 91.1% of them underwent a gastric sleeve to treat their obesity.

Regarding menstruation and fertility before and after surgery, 41.4% had a regular cycle before the operation, which increased to 60.6% after the operation. Also, 48.5% had a normal menstrual flow before the operation, which increased to 61.1% after the operation. 15.2% of the females conceived once before the operation, which barely increased to reach 16.2% after the operation. Additionally, the number of one-time abortions decreased from 11.1% to 7.1% after surgery, and for those who had two abortions, it decreased after the operation from 7.1% to 2%.

In the present study, for the symptoms before surgery, the incidence of having an abnormal menstrual flow, as well as an irregular cycle, were seen significantly more in PCOS patients than non-PCOS (p-value=0.019 and 0.004, respectively). After surgery, the incidence of the normal menstrual flow and the regular menstrual in patients with PCOS had increased (p-value=0.044 and 0.004, respectively). In addition, nearly half of the patients reported their PCOS symptoms and fertility had also improved (55.6% and 47.5%, respectively), and this improvement was statistically significant (p-value=0.031 and 0.043, respectively).

Bariatric surgeries have been evaluated in PCOS patients in different settings. Dilday et al. investigated the outcomes of sleeve gastrectomy in patients with PCOS related to weight loss and fertility. Through 119 PCOS patients, they demonstrated that most of the PCOS patients became pregnant after the operation, with an effective weight loss and weight control [16].

The observations of the latter study support the present study, where 15.2% of the females conceived once before the operation, which increased to 16.2% after the operation. Also, 47% of the whole cohort found that their fertility has improved. Additionally, PCOS symptoms and fertility significantly improved after surgery.

Furthermore, Bhandari et al. evaluated the changes in the fertility hormone in patients with PCOS after sleeve gastrectomy. They included 75 PCOS patients who had a sleeve gastrectomy to treat obesity and revealed that the symptoms of PCOS and levels of fertility hormones showed significant improvement after surgery [17].

These findings of Bhandari et al. are compliant with the outcomes of the present study, where patients with PCOS after surgery had a significantly higher incidence of having a normal menstrual flow and a regular cycle than before the operation [17]. Notably, 91.1% of the female participants in the current study went through sleeve gastrectomy.

Wang et al. examined the use of sleeve gastrectomy as a treatment for PCOS patients through 24 obese patients with PCOS. They reported a significant reduction in androgen levels postoperatively, which resulted in significant improvement in PCOS symptoms [18]. Although the present study did not measure hormonal levels, PCOS symptoms improved after surgery in 55.6% of the females, while 47.5% reported that their fertility has improved.

The present study had some limitations; the study included patients from one city in Saudi Arabia. Furthermore, the study outcomes depend mainly on the responses of the females, which rely on their honesty and subjective opinion, which might affect the reliability of the findings. This is the first study to evaluate the impact of sleeve gastrectomy on PCOS symptoms in Riyadh, Saudi Arabia.

## Conclusions

Sleeve gastrectomy has shown favorable outcomes in patients with PCOS. It can improve symptoms of PCOS in terms of flow and regularity of menstruation, conception, decreasing the incidence of abortion, and improve fertility.

These promising outcomes should provide clinicians with a guide to encourage their female patients towards the surgical treatment of obesity when indicated, in order to improve PCOS and fertility. Further studies in other areas in Saudi Arabia are required. Also, studies evaluating the impact of other bariatric surgeries on PCOS symptoms are encouraged.
